# The *CYLD* p.R758X worldwide recurrent nonsense mutation detected in patients with multiple familial trichoepithelioma type 1, Brooke-Spiegler syndrome and familial cylindromatosis represents a mutational hotspot in the gene

**DOI:** 10.1186/s12863-016-0346-9

**Published:** 2016-02-09

**Authors:** Katalin Farkas, Barbara Kocsis Deák, Laura Cubells Sánchez, Ana Mercedes Victoria Martínez, Juan José Vilata Corell, Alfredo Montoro Botella, Goitzane Marcaida Benito, Raquel Rodríguez López, Tomas Vanecek, Dmitry V. Kazakov, Joan N. R. Kromosoeto, Ans M. W. van den Ouweland, János Varga, Márta Széll, Nikoletta Nagy

**Affiliations:** MTA-SZTE Dermatological Research Group, University of Szeged, Szeged, Hungary; Department of Medical Genetics, University of Szeged, 4 Somogyi B., H-6720 Szeged, Hungary; Department of Dermatology and Venereology of Consorcio Hospital Universitario de Valencia, Valencia, Spain; Genetics Laboratory of Clinical Analysis, Consorcio Hospital General Universitario de Valencia, Valencia, Spain; Unit of Molecular Genetics, Bioptical Laboratory, Pilsen, Czech Republic; The Sikl Department of Pathology, Charles University Medical Faculty Hospital, Medical Faculty in Pilsen, Charles University in Prague, Pilsen, Czech Republic; Department of Clinical Genetics, Erasmus MC, Rotterdam, Netherlands; Department of Dermatology and Allergology, University of Szeged, Szeged, Hungary

**Keywords:** Multiple familial trichoepithelioma type 1, Familial cylindromatosis, Brooke-Spiegler syndrome, Worldwide recurrent mutation, Haplotype analysis

## Abstract

**Background:**

Multiple familial trichoepithelioma type 1 (MFT1; MIM 601606), a rare monogenic skin disease with autosomal dominant inheritance, is characterized by the development of multiple skin-colored papules on the central area of the face, frequently occurring in the nasolabial area. The disease is associated with various mutations in the *cylindromatosis (CYLD;* MIM 605018*)* gene that are also responsible for familial cylindromatosis (FC) and Brooke-Spiegler syndrome (BSS).

**Methods:**

Recently we have identified a Spanish MFT1 pedigree with two affected family members (father and daughter). Direct sequencing of the *CYLD* gene revealed a worldwide recurrent heterozygous nonsense mutation (c.2272C/T, p.R758X) in the patients.

**Results:**

This mutation has already been detected in patients with all three clinical variants – BSS, FC and MFT1 – of the *CYLD*-mutation spectrum. Haplotype analysis was performed for the Spanish patients with MFT1, Dutch patients with FC and an Austrian patient with BSS, all of whom carry the same heterozygous nonsense p.R758X CYLD mutation.

**Conclusions:**

Our results indicate that this position is a mutational hotspot on the gene and that patients carrying the mutation exhibit high phenotypic diversity.

**Electronic supplementary material:**

The online version of this article (doi:10.1186/s12863-016-0346-9) contains supplementary material, which is available to authorized users.

## Background

Multiple familial trichoepithelioma type 1 (MFT1; MIM 601606) is an autosomal dominant condition characterized by numerous firm skin-colored papules that are trichoepitheliomas (follicular tumors). The tumors grow slowly in size and number throughout life, often producing significant cosmetic disfigurement.

MFT1, familial cylindromatosis (FC; MIM 132700) and Brooke-Spiegler syndrome (BSS, MIM 605041) have been independently mapped to chromosome 16q12-q13 by several groups [1–3]. First, FC was mapped to this region in 1995 [1], and its candidate gene, the CYLD gene, was identified in 2000 [3]. Later BSS was mapped to the same region in 2000 [4]. In the mapped region, the same causative gene was identified in 2002 [5]. Regarding MFT1, the same causative gene was identified in 2003 [6]. These genetic investigations supported the previous clinical hypothesis, that MFT1 and FC might be the consequence of the dysfunction of the same gene, since their clinical symptoms can occur in the same patient or in different patients within the same family [7].

In the mapped region, the *cylindromatosis* (*CYLD*) gene [NM_015247] was identified as the causative gene responsible for the development of these three diseases [3]. The gene encodes an enzyme with deubiquitinase activity, which is involved in the post-translational modification of its target proteins and removes Lys63-linked ubiquitin chains [8]. The protein interacts with and negatively regulates the TRAF2, TRAF6, NEMO and BCL3 proteins, affecting the NF-ĸB signaling pathway [8].

Here we report a Spanish MFT1 pedigree with an affected father and daughter, in whom we have identified the recurrent p.R758X CYLD mutation. Previously reported cases carrying the same mutation are reviewed to compare the reported clinical phenotypes and to determine the geographical distribution of the mutation. Moreover, haplotype analysis of the Spanish patients with MFT1, as well as Dutch patients with FC and an Austrian patient with BSS was performed to investigate whether the same or different mutational events are responsible for the development of these cases.

## Methods

### Patients

**Fig. 1:**
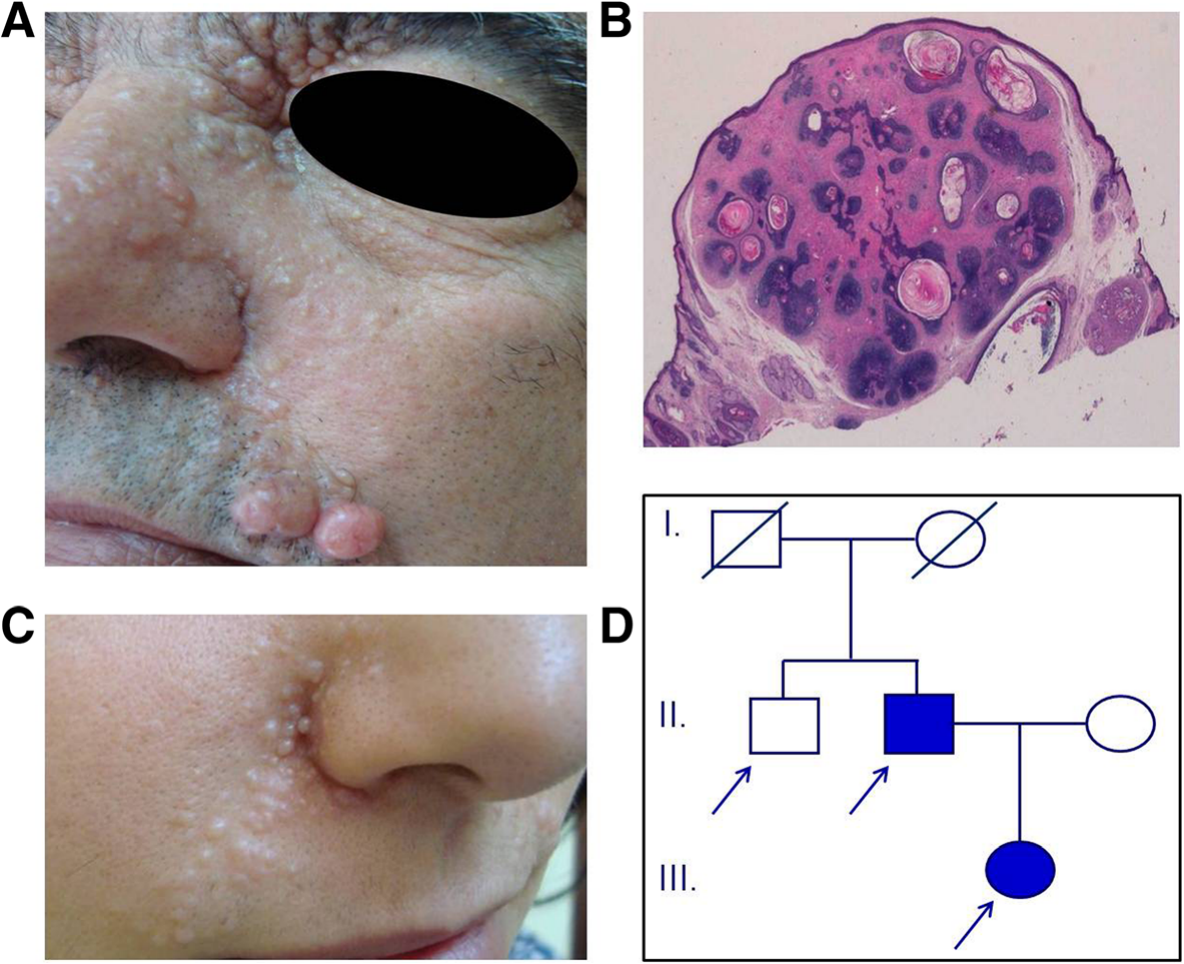
Skin symptoms, histology findings and pedigree of a Spanish family with multiple familial trichoepithelioma type 1. Two affected family members were identified. **a** The father presented skin-colored papules in the periorbital region, nose, nasolabial folds and upper lip. **b** Histological features of trichoepithelioma were islands of basaloid cells with peripheral palisading and small horny cysts (hematoxylin-eosin staining; original magnification × 10). **c** The daughter presented similar but smaller and fewer lesions in the nasolabial fold. **d** The pedigree of the investigated family. Written informed consent was obtained from all participants to publish this article and its accompanying images

The Spanish MFT1 pedigree of Hispanic origin reported here was identified in the Levant region of Valencia, Spain. The 62-year-old father exhibited skin lesions that developed progressively on the central area of the face since the age of 14. Physical examination revealed multiple skin-colored papules measuring a few millimeters and coalescing to form plaques in both nasolabial folds (Fig. 1a), on the forehead, above the eyebrows and, to a lesser extent, on the ears, on the back of the head and on the back. Histological examination of one of the lesions from the right eyebrow revealed multiple basaloid cell aggregates with small keratinized cystic spaces surrounded by specific follicular stroma (Fig. 1b). These findings were consistent with the diagnosis of trichoepithelioma (cribriform trichoblastoma). Subsequently several biopsies were taken and all histological results supported the diagnosis of trichoepithelioma. The patient has been followed for 24 years, during which time the lesions have increased in number and size. Treatment consisted of block excision of multiple localized trichoepithelioma plaques in the nasolabial fold. Other lesions were treated mainly with electrocoagulation. The patient is currently receiving CO_2_ laser treatment.

The patient’s only child, a 33-year-old daughter, has lesions similar to those of her father but are fewer in number (Fig. 1c). The lesions first appeared in both nasolabial folds and, over time, began to appear on her forehead, temples, ears and scalp. Trichoepithelioma was confirmed with a biopsy of a lesion from the right temple. The daughter has been treated with electrocoagulation and cryotherapy, followed by 5 % imiquimod cream. Trichoepitheliomas in the left nasolabial fold were also treated with a single session of photodynamic therapy which had to be discontinued after a few minutes due to intense pain the area. A burn subsequently appeared in the treated area and took weeks to heal. Trichoepitheliomas were resolved but new lesions subsequently appeared in the same area. At present, the patient is receiving a CO_2_ laser treatment and is exhibiting good tolerance and acceptable aesthetic results. To date, no clinical or histological evidence for cylindromas or spiradenomas has been observed in either the father or the daughter. No other clinically affected member has been identified in this pedigree (Fig. 1d).

The data for Dutch and Austrian patients is publically available, and we did not take any samples from these patients ourselves. The investigated Dutch patients were previously reported by Van den Ouweland et al. [9]. Based on the development of cylindromas, these patients were diagnosed with FC. Their detailed clinical description is present in the report of Van den Ouweland et al. [9]. The investigated Austrian patient was previously reported by Grossmann et al. [10]. Based on the development of different skin appendage tumors, the diagnosis of BSS was established. The detailed clinical description of the symptoms is available in the study of Grossmann et al. [10].

### Genetic investigation

The enrollment of the Spanish patients into the genetic investigations have been approved by the Clinical Research Ethics Committee (CEIC) of Consorcio Hospital General Universitario de Valencia. The performed genetic investigation was approved by the Internal Review Board of the University of Szeged, Szeged, Hungary. Written informed consents have been obtained from all the investigated subjects. The study was conducted according to the Principles of the Declaration of Helsinki.

Blood samples for genetic analyses were taken from the Spanish patients and from their clinically unaffected family members, as well as from unrelated controls. Genomic DNA was isolated with a BioRobot EZ1 DSP Workstation (QIAGEN; Godollo, Hungary). The coding regions of the *CYLD* gene and the flanking introns were amplified and sequenced (primer sequences were obtained from the UCSC Genome Browser, www.genome.ucsc.edu).

For haplotype analysis of the Spanish, Dutch and Austrian patients, common polymorphisms (*n* = 33) were genotyped from regions upstream (*n* = 20; rs199912760, rs375106322, rs201860550, rs149502055, rs376795685, rs144013604, rs75157714, rs201233994, rs200973965, rs77528321, rs146702654, rs6145827, rs3064638, rs73584492, rs190892314, rs200678983, rs76797023, rs77678929, rs376799359, rs201103123) and downstream (*n* = 13; rs370702435, rs10451132, rs201757487, rs137990687, rs368656359, rs149201712, rs185111122, rs146946436, rs141129479, rs72796392, rs111543527, rs11866167, rs35072258) of the identified mutation. A detailed list of the investigated polymorphisms is presented in Table 1. Genotypes of the investigated polymorphisms were determined with direct sequencing.

**Table 1:** Haplotype analysis of Spanish, Dutch and Austrian patients carrying the same recurrent nonsense mutation

Nationality		Spanish			Dutch		Austrian
		II/1	II/2	III/1			
		Healthy	Symptomatic	Symptomatic	Symptomatic	Symptomatic	Symptomatic
Polymorphism ID	Frequent allele						
rs35072258	TC/-	TCTC	TCTC	TCTC	TCTC	TCTC	TCTC
rs11866167	C/A	CC	CC	CC	CC	CC	CC
rs111543527	T/C	TT	TT	TT	TT	TT	TT
rs72796392	T/C	TT	TT	TT	TT	TT	TT
rs141129479	A/G	AA	AA	AA	AA	AA	AA
rs146946436	A/G	AA	AA	AA	AA	AA	AA
rs185111122	T/C	TT	TT	TT	TT	TT	TT
rs149201712	AC/-	ACAC	ACAC	ACAC	ACAC	ACAC	ACAC
rs368656359	G/A	GG	GG	GG	GG	GG	GG
rs137990687	G/A	GG	GG	GG	GG	GG	GG
rs201757487	G/-	GG	GG	GG	GG	GG	GG
rs10451132	G/T	GG	GG	GG	GG	GG	GT
rs370702435	A/G	AA	AA	AA	AA	AA	AA
rs121908388	C/T	CC	CT	CT	CT	CT	CT
rs199912760	G/A	GG	GG	GG	GG	GG	GG
rs375106322	G/A	GG	GG	GG	GG	GG	GG
r201860550	G/T	GG	GG	GG	GG	GG	GG
rs149502055	C/T	CC	CC	CC	CC	CC	CC
rs376795685	G/A	GG	GG	GG	GG	GG	GG
rs144013604	A/G	AA	AA	AA	AA	AA	AA
rs75157714	G/A	GG	GG	GG	GG	GG	GG
rs201233994	AT/-	AT	AT	AT	AT	AT	AT
rs200973965	ATAC/-	ATAC	ATAC	ATAC	ATAC	ATAC	ATAC
rs77528321	T/C	TT	TT	TT	TT	TT	TT
rs146702654	T/-	TT	TT	TT	TT	TT	TT
rs6145827	ACACAC/-	ACACAC ACACAC	ACACAC ACACAC	ACACAC ACACAC	ACACAC ACACAC	ACACAC ACACAC	ACACAC ACACAC
rs3064638	ACACAC/-	ACACAC ACACAC	ACACAC ACACAC	ACACAC ACACAC	ACACAC ACACAC	ACACAC ACACAC	- -
rs73584492	A/G	AA	AA	AA	AA	AA	AA
rs190892314	A/G	AA	AA	AA	AA	AA	AA
rs200678983	C/T	CC	CC	CC	CC	CC	CC
rs76797023	A/T	AA	AA	AA	AA	AA	AA
rs77678929	T/A	TT	TT	TT	TT	TT	TT
rs376799359	A/T	AA	AA	AA	AA	AA	AA
rs201103123	C/T	CC	CC	CC	CC	CC	CC

## Results and discussion

**Fig. 2:**
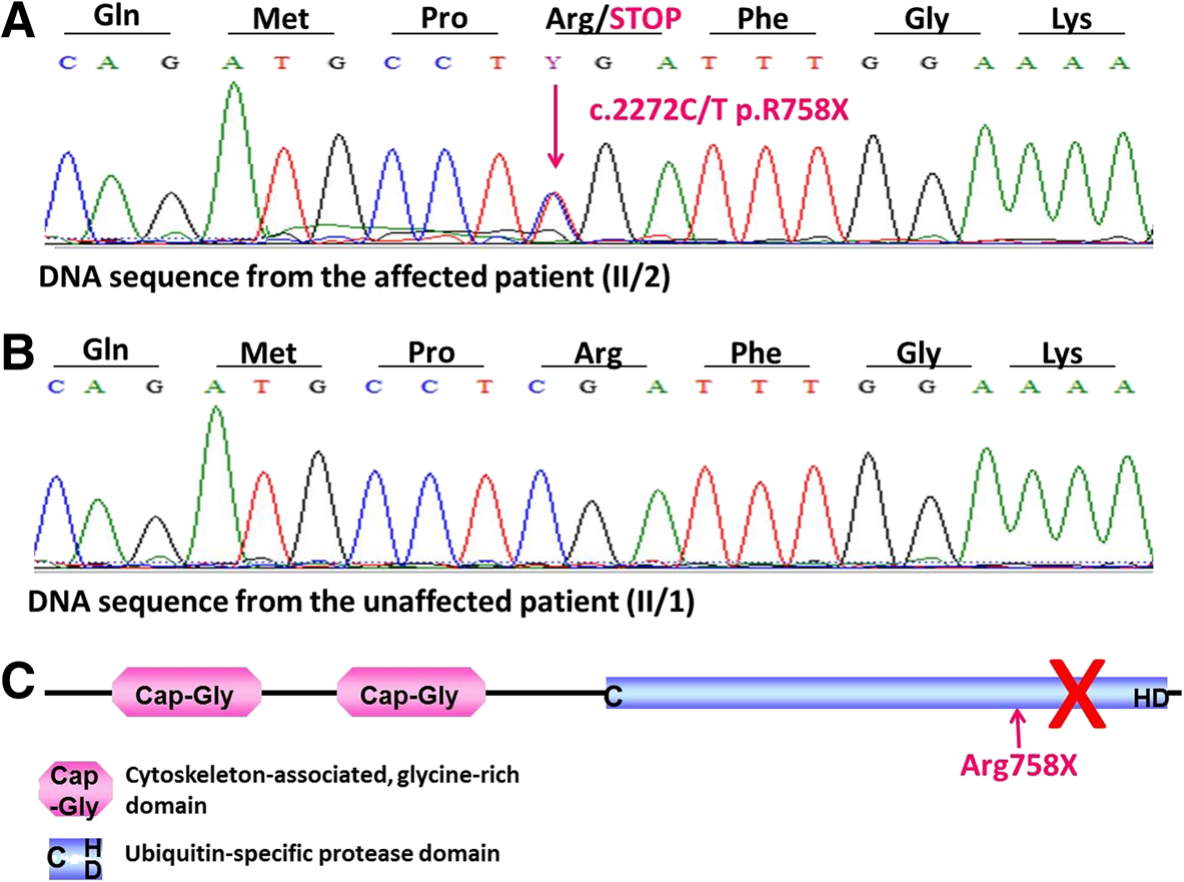
Direct sequencing of the *CYLD* gene. Direct sequencing revealed a nonsense mutation (c.2272C/T, p.R758X) in exon 17. **a** The affected family members carried the deletion in heterozygous form. **b** The unaffected family members carried the wild-type sequence. **c** The mutation is located in the region of the ubiquitin-specific protease domain of the CYLD protein

Direct sequencing of the coding regions and the flanking introns of the *CYLD* gene from the investigated Spanish patients revealed a previously described nonsense mutation in exon 17 (c.2272C/T, p.R758X, rs121908388). This mutation results in a premature termination codon causing truncation and, thus, dysfunction of the CYLD protein. Both patients carried the mutation in heterozygous form (Fig. 2a), whereas the unaffected family members and unrelated controls carried the wild-type sequence (Fig. 2b, Additional file 1.). The identified mutation was located in the ubiquitin-specific protease domain of the CYLD protein (Fig. 2c).

Previously reported Dutch and Austrian cases carrying the same mutation were also investigated in this study [9, 10]. Haplotype analysis of the Spanish patients with MFT1, the Dutch patients with FC and Austrian patients with BSS was performed (Table 1.). Our results demonstrated that the Spanish and the Dutch pedigrees carry the same haplotype, whereas the Austrian patient carries a different haplotype. Thus, it can be assumed that different mutational events are responsible for the development of the Austrian case and the Spanish and Dutch cases.

A review of all previous studies reporting the same c.2272C/T, p.R758X nonsense mutation of the *CYLD* gene revealed that this mutation has also been detected in patients with BSS [10–12], FC [3, 9, 13] and MFT1 [10, 14]. Thus, the c.2272C/T, p.R758X nonsense mutation of the *CYLD* gene can lead to the manifestation of any of the clinical variants in the disease spectrum caused by *CYLD* mutation, which is associated with high phenotypic diversity. Furthermore, this mutation has been detected in Caucasian American [3], South African [10], Austrian [10, 14], Czech [11], Dutch [9], Chinese [12] and Japanese patients [13] and is, thus, considered a recurrent worldwide mutation (Table 2.). These data suggest that the c.2272C/T, p.R758X nonsense mutation is located at a mutational hotspot in the *CYLD* gene.

**Table 2:** Summary of the geographical location and clinical manifestation of the recurrent p.R758X *CYLD* mutation

CYLD cDNA	CYLD protein	Detected in patients with	Nationality	References
c.2272C > T	p.R758X	FC	Caucasian American, Dutch, Japanese	[3, 9, 13]
		BSS	Austrian, South African, Czech, Chinese	[10–12]
		MFT1	Austrian, Spanish	[10, 14], present study

To determine whether the worldwide recurrent p.R758X mutation of the *CYLD* gene is the result of one or more independent mutational events, we performed haplotype analysis. The haplotype analysis of the Spanish, Dutch and Austrian patients demonstrated that, although the Spanish and the Dutch patients carry the same haplotype, the clinical appearance, MFT1 and FC, respectively, is different (Table 1.). These results suggest the importance of modifying genetic and/or environmental factors. In contrast with these, the Austrian patient carried a different haplotype than the Spanish and Dutch families. Thus, we assume the presence of the same mutation is the consequence of different mutational events (Table 1.).

## Conclusion

Our results support the conclusion that position 2272 in the nucleotide sequence of the *CYLD* cDNA [NM_015247] is a mutational hotspot on the *CYLD* gene. This result correlates well with our previous findings for Hungarian and Anglo-Saxon BSS families carrying the same heterozygous nonsense mutation (c.2806C > T, p.Arg936X) but different haplotypes [15]. Of note, both mutational hotspots are the location of recurrent nonsense mutations. Regarding the encoded functional domains, both of mutational hotspots affect the ubiquitin-specific protease domain of the CYLD protein (Fig. 2c). Both recurrent nonsense mutations have been reported for all three clinical variants (MFT1, BSS, FC) of the *CYLD*-mutation based disease spectrum and have been associated with high intra- and interfamilial phenotypic diversity [15].

These reports raise the question of how these two worldwide recurrent nonsense mutations can lead to the development of the different clinical variants of the CYLD-mutation based disease spectrum. Further studies are needed to identify putative genetic, environmental or lifestyle factors and to elucidate the mechanism leading to the enormous phenotypic differences observed in patients carrying the same c.2272C/T, p.R758X nonsense mutation.

### Availability of supporting data

All the supporting data are included as additional file.

### Consent to publish

Written informed consent was obtained from all participants to publish this article.

## Additional file

Additional file 1:
**Sequencing data of Patient III/1 demonstrated the same heterozygous mutation, which has been detected in case of Patient II/2.** (JPG 163 kb)

## Abbreviations

MFT1: Multiple familial trichoepithelioma type 1
FC: familial cylindromatosis
BSS: Brooke-Spiegler syndrome
CYLD: cylindromatosis gene
TRAF2: TNF receptor-associated factor 2
TRAF6: TNF receptor-associated factor 6
NEMO: NF-kappa-B essential modulator
BCL3: B-cell lymphoma 3-encoded protein
NF-κB: nuclear factor kappa-light-chain-enhancer of activated B cells
