# Supplementary figures and images for: The CYLD p.R758X worldwide recurrent nonsense mutation detected in patients with multiple familial trichoepithelioma type 1, Brooke-Spiegler syndrome and familial cylindromatosis represents a mutational hotspot in the gene
Source: BMC Genet. 2016 Feb 9;17:36. doi: 10.1186/s12863-016-0346-9 (PMC4746830; doi:10.1186/s12863-016-0346-9)

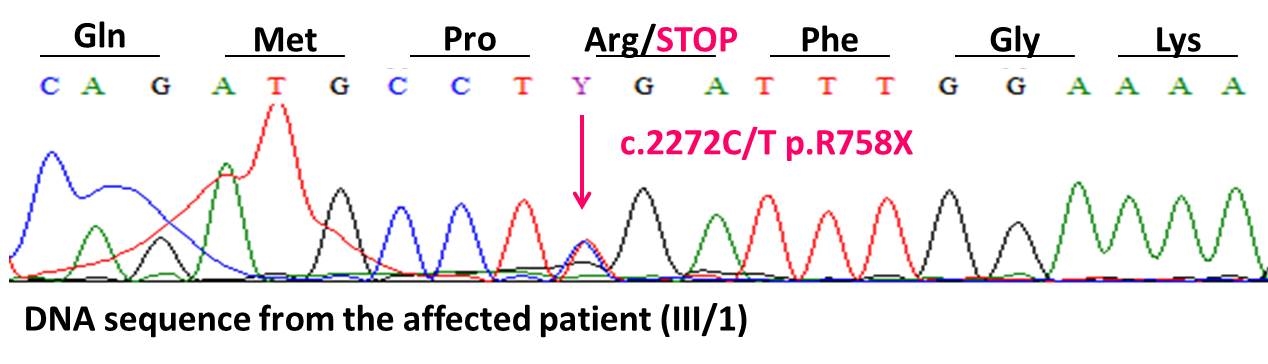

Supplement: Additional file 1: — Sequencing data of Patient III/1 demonstrated the same heterozygous mutation, which has been detected in case of Patient II/2. (JPG 163 kb) [file 12863_2016_346_MOESM1_ESM.jpg]
